# Says Blood Will Prove a Man’s Race

**Published:** 1913-10-15

**Authors:** 


### SAYS BLOOD WILL PROVE A MAN'S RACE.

---

Prof. Edward T. Reichert, of the University of Pennsylvania, in a report just made to the Carnegie Institution, at Washington, on his researches to discover if possible a method of producing life artificially, has announced the discovery of the blood characteristics of various human races.

Dr. Reichert declares that as soon as he has completed his experiments he will be able to differentiate between the blood of a Chinaman, Indian, negro and other races so as to make his discovery of absolute value in a diagnostic way for medico-legal work.

By a study of the different blood cells Dr. Reichert says he has found that it is absolutely impossible to mistake the blood crystals of one animal for those of another, just as it would be impossible to mistake the animals themselves.

Dr. Reichert describes his discovery by enumerating some of the animals that he has reclassified. In the old method of classifying animals according to their tribes the bear was always placed in the same family as the dog, the wolf and the fox.

By the new method of comparing blood crystals of those animals, Dr. Reichert has proved to his satisfaction that the bear is related closely to the sea lions and the seals, as naturalists have contended, and is not related to the dog, the wolf or the fox.

In the bird world Dr. Reichert says the guinea foul has been c'assed as belonging to the same family as the domestic fowl. He has proved also to his satisfaction that the guinea fowl's blood crystals are like the ostrich's.

In his preliminary researches Dr. Reichert studied plant life to get at the underlying methods of the formation of the protoplasm. He says that starches from different plants vary in their physical and physio-chemical properties, and that the differences are distinctive of the plant and can be plotted out in the form of reaction curves which show the species and the genera from which they spring.

His laboratory records contain a description of 1,200 starches, obtained from a large number of plants and plant parts from all over the world. The scientist declares that the difference in the properties of the complex organic metabolic plants and animals offers a logical basis for the reclassification of animals according to the forms of cell life with which they were born.

Dr. Reichert believes he has arrived at the very mechanisms which give rise to the phenomena which in the aggregate constitute life.—*Dental Scrap Book.*

